# Self-report versus performance based executive functioning in people with psychotic disorders

**DOI:** 10.1016/j.scog.2023.100293

**Published:** 2023-10-20

**Authors:** B.C. van Aken, R. Rietveld, A.I. Wierdsma, Y. Voskes, G.H.M. Pijnenborg, J. van Weeghel, C.L. Mulder

**Affiliations:** a Department of Psychiatry, Erasmus MC, Epidemiological and Social Psychiatric Research Institute, Rotterdam, the Netherlands; b Fivoor Forensic Psychiatric Institute, Rotterdam, the Netherlands; c Department of Ethics, Law and Humanities, Amsterdam UMC, Amsterdam, the Netherlands; d GGz Breburg, Tilburg, the Netherlands; e Department of Psychotic Disorders, GGZ Drenthe, Assen, the Netherlands; f Department of Clinical and Developmental Neuropsychology, Faculty of Behavioral and Social Sciences, University of Groningen, Groningen, the Netherlands; g Phrenos Centre of Expertise, Utrecht, the Netherlands; h Tranzo Department, Tilburg School of Behavioural and Social Sciences, Tilburg University, Tilburg, the Netherlands; i Parnassia Psychiatric Institute, Rotterdam, the Netherlands

**Keywords:** Executive functioning, Performance based, Self-report

## Abstract

**Background:**

Although executive functioning is often measured using performance-based measures, these measures have their limits, and self-report measures may provide added value. Especially since these two types of measures often do not correlate with one another. It thus has been proposed they might measure different aspects of the same construct. To explore the differences between a performance-based measure of executive functioning and a self-report measure, we examined their associations in patients with a psychotic disorder with the following: other neurocognitive measures; psychotic symptoms; anxiety and depression symptoms, and daily-life outcome measures.

**Method:**

This cross-sectional study consisted of baseline measures collected as part of a cohort study of people with a psychotic disorder (the UP'S study; *n* = 301). The Behavioral Rating Inventory of Executive Functioning Adult version (BRIEF-A) was used to assess self-rated executive functioning, and the Tower of London (TOL) to assess performance-based executive functioning. Generalized linear models (GLM) were used with the appropriate distribution and link function to study the associations between TOL and BRIEF-A, and the other variables, including the Brief Assessment of Cognition in Schizophrenia (BACS), the Positive and Negative Symptoms Scale-Remission (PANSS-R), the General Anxiety Disorder – 7 (GAD-7), the Patient Health Questionnaire – 9 (PHQ-9) and the WHO Disability Assessment Schedule 2.0 (WHODAS 2.0). Model selection was based on the Wald test.

**Results:**

The TOL was associated with other neurocognitive measures, such as verbal list learning (β = 0.24), digit sequencing (β = 0.35); token motor task (β = 0.20); verbal fluency (β = 0.24); symbol coding (β = 0.43); and a screener for intelligence (β = 2.02). It was not associated with PANNS-R or WHO-DAS scores. In contrast, the BRIEF-A was associated not with other neurocognitive measures, but with the PANSS-R (β = 0.32); PHQ-9 (β = 0.52); and GAD-7 (β = 0.55); and with all the WHODAS domains: cognition domain (β = 0.54), mobility domain (β = 0.30) and selfcare domain (β = 0.22).

**Conclusion:**

Performance-based and self-report measures of executive functioning measure different aspects of executive functioning. Both have different associations with neurocognition, symptomatology and daily functioning measures. The difference between the two instruments is probably due to differences in the underlying construct assessed.

## 1 Introduction

Cognitive impairments are thought to be core features of psychosis (Velligan and Bow-Thomas, 1999; Kahn and Keefe, 2013; Keefe and Harvey, 2012; Holmén et al., 2012). Although they are present before the onset of the illness (Mollon and Reichenberg, 2018; Dickson et al., 2012; Jahshan et al., 2010) and worsen during the first episode (Sheffield et al., 2018; Tempelaar et al., 2017; Barder et al., 2013; Freedman and Brown, 2011), they often seem to stabilize thereafter (Fucetola et al., 2000; Bowie et al., 2008; Kurtz, 2005**).** People diagnosed with a psychotic disorder nonetheless perform worse on cognitive tests compared to healthy controls, the overall performance of those diagnosed with schizophrenia being the worst (Zanelli et al., **2010**). In this regard, the executive functions are among the most impacted cognitive skills (Meier et al., 2014; Liu et al., 2011; Chan et al., 2012).

While executive functioning is a collective term for distinct cognitive processes (Miyake et al., 2000), there is no consensus on an exact definition of executive functions (EF). We nonetheless know from overlaps between the wide range of competing definitions (Barkley, 2012; Goldstein and Naglieri, 2014) that executive functions help us to interact with the world around us and to respond to novel and/or demanding situations, thereby helping us to attain goals by adjusting our behavior according to inputs from the environment and those around us (Barkley, 2012). Executive functions are thus goal-directed processes involved in active real-time decision-making, planning, self-control, and initiating behavior (Frangou, 2010; Coulacoglou and Saklofske, 2017; Kerns et al., 2008; van der Stel et al., 2015).

Due to this absence of an overall accepted definition, there are also various ways of measuring executive functioning, the most common involving performance-based measures. As these are structured observatory tests, they are thought to capture executive functions well. However, even though much of what is seen as executive functioning involves human actions in daily settings (Barkley, 2012; Delis, 2014), these tests were created and used within research labs (Gohier et al., 2009), and often measure only one form of executive functioning (Miyake et al., 2000). As a result, they often have limited ecological validity (Ardila, 2008; Wilson, 1993). Neither do they correlate with other valid EF ratings, such as self-report or teacher ratings (Toplak et al., 2013). They also share limited variance with ratings of daily self-care and adaptive functioning (Alderman et al., 2003; Barkley and Fischer, 2011).

To complicate matters further, these tests not only have limited value in predicting executive functioning in daily life, they also measure multiple general cognitive skills at the same time (Barkley, 2011; Collette et al., 2006), making it hard to identify distinct executive functions. For example, a lab task often demands not just task execution, but also goal identification and problem-solving strategies. If any of these processes is impaired, the task will not specify which impairment is present (Köstering et al., 2015; Snyder et al., 2015), because a simplified task is underspecified to tap into the complex cognitive processes entailed in executive functioning (Berg et al., 2010; Kaller et al., 2011; Miyake and Friedman, 2012). As a consequence, these tests may not be the most effective for discriminating between patients and healthy controls (Snyder et al., 2015; Weyandt et al., 2014).

Another way of testing executive functioning is by using self-report measures. While these can provide insight into executive functioning in everyday life (Gioia, 2008; Payne et al., 2011), they also have limitations. They may provide a less specific portrait of impairments in executive functioning because they may be less separable in a daily context (Naglieri and Goldstein, 2014). For example: during studying, an inability to inhibit distractions might overlap with problems in working memory, making it difficult to determine which executive function results in difficulty with studying. Ratings may also be affected by environmental and psychological factors, over which there is limited control. Someone in a highly demanding environment might rate themselves lower than someone in a simpler, less demanding environment, whose executive functions may be not better per se (Chaytor et al., 2006). Furthermore, emotional state or personality trait might influence the ratings both negatively or positively (Kajonius and Björkman, 2020; Buchanan, 2016), whereas impaired insight (Burton et al., 2016; Saperstein et al., 2012; Poletti et al., 2012) and metacognition (Butzbach et al., 2021; Ciurli et al., 2010) often result in poor ratings of cognition.

Given the differences between them, it is not surprising that performance-based and self-report measures are not strongly correlated measures of executive functioning (Meltzer et al., 2017; Nordvall et al., 2017; Haugen et al., 2021), even though both have been validated for that purpose. This could reflect differences in validity between the two measures. For example, when self-reported measures are used as an addition to performance-based measures, they are thought to add ecological validity (Gioia et al., 2010). On the other hand, performance-based measures are thought to add discriminant validity to self-report measures (Isquith et al., 2014). A lack of correlation may also reflect a difference in the measured constructs of executive functioning (Nordvall et al., 2017; Ten Eycke and Dewey, 2016). Previous research has suggested that performance-based measures tap into processing efficiency and cognitive control, whereas self-report measures are thought to measure the accomplishment of goal pursuit in real life (Toplak et al., 2013; Nordvall et al., 2017). In short, performance-based measures are thought to reflect the technical efficiency of the executive functions, whereas self-report measures might better reflect how the executive functions can be used successfully in daily life.

Either way, the lack of correlation between performance-based and self-report executive functioning indicates that executive functions could best be measured using multiple forms of testing, as has been recommended (Isquith et al., 2013; Goldstein et al., 1993; Prouteau et al., 2004). However, nearly all research that has used multiple forms of executive functioning measurements was done in populations with ADHD, Traumatic Brain Injuries or other frontal-lobe impairments (Weyandt et al., 2014). No such comparisons between different forms of executive functioning testing have been made in psychotic-disorder populations. An additional difficulty is that most psychosis research still uses only performance-based measures (Miyake et al., 2000; Gohier et al., 2009) to assess associations with e.g. clinical or functional outcomes such as symptoms or work or study. This means that we do not know how self-report executive function measures correlate with symptomatologic measures or daily functioning outcomes. Furthermore, we do not know whether the lack of correlation between the performance-based and self-report measures reflects a different ecological validity or a difference in underlying constructs regarding executive functioning. We explore the difference between performance-based and self-report measures of executive functions by comparing both measures to other neurocognitive measures, symptomatology measures and a daily life outcome measure. By doing so, we hope to find a clear overview of how both measures relate to these different important outcomes. This overview can bring us one step further on whether the underlying construct of the two measures are different, or whether the psychometric capacities of the two results in the lack of correlation.

When exploring the differences between performance-based and self-report measures of executive functioning in patients with a psychotic disorder, we therefore analyzed their associations with three sets of other variables. After investigating their associations with other neurocognitive measures, we examined their associations with various symptomatology measures, and then their respective associations with daily-life outcome measures. In all cases, we explored whether there might be an interaction between the performance-based and self-report measures, to further look into the underlying constructs. As in previous research in other populations, we expected that correlations between these two measures of executive functioning would be low. Furthermore, previous research showed that there is reason to believe that performance-based measures reflect the technical efficiency of the executive functions, whereas self-report measures might better reflect how the executive functions can be used successfully in daily life. This is why we expect that performance-based measures would show stronger associations with other performance-based neurocognitive measures; and, in contrast, that the self-report measure would be associated with symptoms and daily-life outcome measures.

## 2 Methods

The UP'S study is an observational cohort intended to examine processes of recovery in people with psychotic disorders over a 10-year period (van Aken et al., 2021; Mulder and van Aken, 2021). In a collaboration between Erasmus University Medical Centre and mental health care institutions in the southwestern Netherlands, each institution provides ambulatory teams in which eligible clients can be included. Such clients have a primary diagnosis of a schizophrenia spectrum disorder according to the DSM 5 criteria (i.e., schizophrenia, schizophreniform disorder, schizoaffective disorder, brief psychotic disorder, substance-induced psychotic disorder, delusional disorder, schizotypical disorder, or psychotic disorder not otherwise specified). At inclusion, they are aged between 18 and 65. Those with insufficient proficiency in Dutch are excluded. All patients are assessed yearly.

Inclusion and baseline interviews are all handled by students or researchers at a participating ambulatory team. Follow-up measurements are handled by students and/or researchers at Erasmus University Medical Centre. To determine who will be asked to participate, an anonymized list is compiled specifying all eligible clients in the team. Per participating team, this draws on the Electronic Patient Files (EPF), and is based on age and primary diagnosis, the latter being determined after a clinical interview with the psychiatrist of the team. Those who are not eligible for inclusion are filtered out.

Thirty patients from the remaining list are then randomly selected. If fewer than 30 patients of the team meet the inclusion criteria, all eligible patients are placed on the list. However, a patient is not contacted if the team considers that he or she may be unable to participate for a specific reason such as active psychosis, or ineligibility for reasons such as legal detention. In other words, the only patients who can be asked to participate are those who were on the list and who, in the team's view, can be approached.

Patients may give informed consent and participate in the study only after they have been given information on the study, have received answers to all their questions, and have been given time to consider participation. Those who are not willing to participate are asked for a reason and will not be contacted again. Unfortunately, no demographic or clinical data is available on patients who are not willing to participate. For this reason, we cannot compare those that were and were not included in the study. This study contains the baseline data on *N* = 301 participants who had been included and who, by April 13th, 2022, had completed the relevant questionnaires.

### 2.1 Measures

#### 2.1.1 Performance-based executive functioning

*The Tower of London (TOL)* is a widely used task designed to score planning and executive functioning. Participants are shown two pictures, each with three sticks that have three beads (red, green, and blue) stacked on them. One picture shows the starting situation, the second shows the end situation. The goal is to calculate the lowest number of steps necessary to go from the first to the second picture (Shallice, 1982). There are 20 basic items and 2 bonus items, which are only shown when all basic items have been answered correctly within 20 s. Therefore, the higher the score, the better the efficiency of a person's executive functioning system. In this cohort study, the TOL is part of the Brief Assessment of Cognition in Schizophrenia (BACS).

#### 2.1.2 Self-reported executive functioning

*The Behavior Rating Inventory of Executive Functioning for Adults (BRIEF-A)* is a 76-item self-report questionnaire designed to assess executive functioning in real-life situations (Roth et al., 2014). Each item is scored on a three-level scale ranging from 1 (never) to 3 (always). Each item is part of one of nine subscales, which in turn are part of two indices: the Behavior Regulation Index (BRI) and the Metacognitive Index (MI). These two indices can be summarized to a total score: the Global Executive Composite score (GEC). For all indices, t-scores and percentiles must be calculated, which can be used to compare scores to different research and populations. In this study, only the GEC was used. Clinically relevant interpretations can be made on the basis of the t-scores and percentiles. T-scores above 65 or a percentile above 90 are considered clinical scores; t-scores between 60 and 65 are subclinical, and t-scores below 60 are considered normal. In this case, the higher a person's score on the BRIEF-A, the worse they are at using their executive functions. The scores can be interpreted only when scores are below cut-off on the three validity scales, i.e., negativity, improbability, and inconsistency.

In this study, all invalid scores were excluded from the analysis. The questionnaire has been evaluated for use in a schizophrenia sample (Power et al., 2012). In this cohort, Chronbach's α for the questionnaire was 0.91.

#### 2.1.3 Neurocognition

*The Brief Assessment of Cognition in Schizophrenia (BACS)* was developed as a tool for measuring cognition in schizophrenia for clinical trials. It contains six subtests, all on domains that are found to be consistently impaired in schizophrenia. The subtests are 1.) list learning for verbal memory, 2.) the digit-sequencing task for working memory, 3.) the token motor task for motor speed, 4.) the semantic category fluency and alphabetical category fluency for verbal fluency, 5.) the symbol coding task for attention and speed of information processing and 6.) the Tower of London for executive functions referred to above. A detailed description of all tests can be found in RS Keefe, TE Goldberg, PD Harvey, JM Gold, MP Poe and L Coughenour (Keefe et al., 2004). Chronbach's α for the entire BACS in this cohort was 0.62.

*The Screener for Intelligence and Learning disabilities (SCIL)* was developed as a quick intelligence-screening method capable of detecting an intellectual disability (IQ 50–85). It has 14-items that assess four different domains: schooling, school skills, social contacts and language comprehension (Nijman et al., 2018). The questions therefore range from the highest level of education to whether someone reads a newspaper and has a support system. It takes only 15 min to complete, and, after training, can be administered by any professional. Since some questions entail school skills such as arithmetic or drawing a clock, sufficient concentration is needed during the test. In this cohort, Cronbach's α for the SCIL was 0.71.

#### 2.1.4 Symptom severity

*The Positive and Negative Symptom Scale – Remission (PANSS-R)* is a short version of the PANSS. The original PANSS is a 30-item inventory to assess psychotic symptom severity. This shortened remission version contains 8 of the 30 items across three subscales: three positive symptoms, three negative symptoms, and two general symptoms (Andreasen et al., 2005). Each item can be scored from 1 (absent) to 7 (extreme), with scores incorporating both the severity of the symptom as well as its behavioral effect (Kay et al., 1987). Both a mean score for each subscale and the total scale are used in the analysis. If one item is missing, the mean of the other six items will be imputed. If more than one item is missing, no subscale or total score can be calculated. In this cohort, Chronbach's α for the PANSS-R was 0.67.

*The Patient Health Questionnaire – 9 (PHQ-9)* is a shortened screener derived from the original version of the PHQ, which contains five modules, each covering one of five mental disorders: depression, anxiety, somatoform, alcohol and eating. This shortened version is the depression module, which contains 9 items scored from 0 (Not at all) to 3 (Nearly every day). A severity sum score can be calculated, ranging from 0 to 27. In this cohort, Chronbach's α for the PHQ-9 was 0.84.

*The Generalized Anxiety Disorder – 7 (GAD-7)* is an anxiety screener which was developed after the PHQ. It contains 7 items, which are scored 0 (Not at all) to 3 (Nearly every day). A severity sum score can be calculated, ranging from 0 to 21. In this cohort, Chronbach's α for the GAD-7 was 0.86.

#### 2.1.5 Daily functioning

*The World Health Organization – Disability Assessment Schedule 2.0 (WHODAS 2.0)* is a self-report questionnaire developed as a standardized way of capturing disability in daily life (World Health O, 2014). Developed on the basis of item-selection of the ICF-10 (Williams et al., 2021), it can be divided into 6 domains: cognition, mobility, selfcare, getting along, life activities and participation. The first three domains entail the dimension of daily activities, which captures limitations in these activities. This dimension is used here as a way to measure disability in daily activities. All questions ask for the perceived disability in the last 30 days and are scored from 1 (None) to 5 (A lot/Unable). Scores are then calculated on the basis of the complex scoring method, which is based on the item-response theory. It takes account of the level of difficulty for each item. The summary score is then converted into a metric range from 0 to 100, where 0 is no disability and 100 is full disability (Williams et al., 2021). In this cohort, Chronbach's α for the WHODAS Daily life domain was 0.66.

### 2.2 Statistical analyses

Demographic sample characteristics and questionnaire scores were summarized either as means and standard deviations or as percentages. Missing values and outliers for all questionnaires were inspected and processed according to questionnaire standards.

Then, to compare the effects of TOL and BRIEF-A, generalized linear models (GLM) were used with the appropriate distribution and link function. We controlled for age and gender on three different types of outcome measures. In the first step, TOL and BRIEF-A scores were associated with neurocognition measures of the BACS, i.e., the list-learning task, digit-sequencing task, token motor task, category instances, oral word-association and symbol-coding task. We also examined the effects on intelligence and learning disability. In the second step, TOL and BRIEF-A scores were associated of the severity of clinical symptoms as measured with the PANSS-R, GAD-7 and PHQ-9. Finally, executive functioning was associated with limitations in daily life activities as measured with the WHODAS. Model selection was based on the Wald test with the critical value set at the conventional 0.05 level. In all cases, an interaction term between the TOL and BRIEF-A was explored as well, since both measures are expected not to correlate. Exploratory sensitivity analyses were planned with the TOL dichotomously rather than continuously. This based on both the skewness of the TOL and the idea that the underlying concept (technical efficiency) might be best reflected by a ‘on’ versus ‘off’ state. Again, interaction terms between the (dichotomous) TOL and the BRIEF-A were explored as well. Given the exploratory nature of this study, no correction for multiple testing was applied. All analyses were performed using SPSS version 27.0 (IBM Corp., Armonk, NY, USA).

## 3 Results

At the time of writing this article, 354 participants had been included in the UP'S cohort study, 301 of whom had finished the baseline interview. Their mean age was 41.7 years (SD = 12.2, range 18–65), and 67.4 % were male. Of the participants, 39.2 % had a primary diagnosis of schizophrenia and 35.4 % had finished secondary vocational education.

The mean score on the TOL was 15.29 (SD = 5.1), which was similar to that in outpatient populations (Wang et al., 2016; Segarra et al., 2011) and better than in inpatient populations with schizophrenia (Keefe et al., 2004; Haddad et al., 2021). The mean score on the BRIEF-A was 57.94 (SD = 11.5), which was marginally better than in other outpatient psychotic disorder patient samples, but still far below that of a healthy population (Bulzacka et al., 2013).

Table 1 shows the mean scores on all measurements, their SD, and their comparisons with other populations. Results for all measures were similar to those for schizophrenic or psychotic (outpatient) populations. The low correlation between the TOL and BRIEF-A (*r =* −0.05, *p* = .447) was similar to that in other research (Haugen et al., 2021).

**Table 1:** Descriptive statistics.

					Population comparison
			Range	Mean (SD)	
Executive functioning	BRIEF-A⁎		35–91	57.94 (11.5)	(Bulzacka et al., 2013)
	TOL		0–22	15.29 (5.1)	(Wang et al., 2016; Segarra et al., 2011)
Neurocognition	BACS	*List learning*	4–64	34.18 (11.9)	(Wang et al., 2016; Segarra et al., 2011)
		*Digit sequencing*	4–28	17.25 (4.9)	
		*Token motor task*	4–100	61.19 (17.3)	
		*Verbal fluency*	0–74	37.34 (11.8)	
		*Symbol coding*	0–78	39.84 (13.7)	
	SCIL		4–28	19.58 (5.1)	(Nieuwenhuis et al., 2017)
Symptoms	PANSS-R Total		1–4.5	1.98 (0.8)	(Brunet-Gouet et al., 2020; Mosolov et al., 2012)
	PHQ-9		0–25	7.58 (5.8)	(Quek et al., 2021; Sj et al., 2021)
	GAD-7		0–20	6.19 (5.14)	(Sj et al., 2021)
Daily functioning	WHO-DAS Daily activity	*Domain 1: Cognition*	10–90	31.63 (18.7)	(Brunet-Gouet et al., 2020; Mosolov et al., 2012)
		*Domain 2: Moving around*	12.5–100	26.70 (19.2)	
		*Domain 3: Self-care*	30–89	33.75 (8.2)	

BRIEF-A = Behavioral Rating Inventory of Executive Functioning – Adults; TOL = Tower of London; BACS = Brief Assessment of Cognition in Schizophrenia; SCIL = Screener of Intelligence and Learning Disability; PANSS-R = Positive and Negative Symptom Severity – Remission; PHQ-9 = Patient Health Questionnaire – 9-item; GAD-7 = Generalized Anxiety Disorder – 7 item; WHO-DAS = World Health Organization – Disability Assessment Schedule; IHS = Integral Recovery Scale (in Dutch: Integrale Herstel Schaal).

⁎ BRIEF-A scores apply only to participants having a valid score according to BRIEF-A guidelines.

### 3.1 Executive functioning and neurocognition

Regression models of the different BACS measures showed statistically significant associations between the TOL and on all different neurocognitive measures and the SCIL. However, except for a small effect on the Token Motor Task (β = −0.14) and on the Symbol Coding (β = −0.13), there were no effects for the BRIEF-A. There was only a small interaction effect between the TOL and BRIEF-A on the verbal list learning (β = −0.15). All results for the selected model are shown in Table 2. Since the TOL was skewed, it seemed necessary to view the efficiency of the EF system more in an on/off sense than as a scale from bad to good. For the sensitivity analysis we therefore performed the same regression models with the TOL dichotomously. The only difference in results concerned the BACS Verbal List Learning, where the interaction between the BRIEF-A and TOL dichotomously had an effect on the Verbal List learning score.

**Table 2:** Regression model of executive functioning on neurocognition.

	Model 1 (BACS -LL)		Model 2 (BACS-DS)		Model 3 (BACS-TMT)		Model 4 (BACS-VF)		Model 5 (BACS-SC)		Model 6 (SCIL)	
Parameter	β (SD)	Wald X^2^	β (SD)	Wald X^2^	β (SD)	Wald X^2^	β (SD)	Wald X^2^	β (SD)	Wald X^2^	β (SD)	Wald X^2^
Intercept	0.02 (0.11)	0.03	−0.19 (0.11)	2.78	0.15 (0.11)	1.78	−0.06 (0.11)	0.30	−0.08 (0.10)	0.56	**19.56 (0.53)**	**1342.67**⁎⁎⁎
Sex (male)	−0.09 (0.14)	0.45	0.24 (0.13)	3.21	−0.21 (0.14)	2.51	0.10 (0.14)	0.51	0.02 (0.12)	0.03	0.18 (0.65)	0.08
Age (centered)	**−0.03 (0.01)**	**28.68**⁎⁎⁎	**−0.01 (0.01)**	**4.28**⁎	**0.02 (0.01)**	**21.25**⁎⁎⁎	−0.01 (0.01)	2.15	**−0.02 (0.01)**	**23.79**⁎⁎⁎	0.00 (0.03)	0.01
TOL	**0.24 (0.06)**	**13.26**⁎⁎⁎	**0.35 (0.06)**	**32.09**⁎⁎⁎	**0.20 (0.06)**	**10.11**⁎⁎	**0.24 (0.06)**	**14.22**⁎⁎⁎	**0.43 (0.06)**	**51.96**⁎⁎⁎	**2.02 (0.30)**	**45.71**⁎⁎⁎
BRIEF-A	0.02 (0.07)	0.09	−0.07 (0.07)	1.03	−0.14 (0.07)	**4.35**⁎	−0.01 (0.08)	0.03	−0.13 (0.07)	**4.27**⁎	−0.40 (0.32)	1.53

TOL = Tower of London; BRIEF-A = Behavioral Rating Inventory of Executive Functioning – Adults; BACS = Brief Assessment of Cognition in Schizophrenia; LL = List Learning; DS = Digit Sequencing; TMT = Token Motor Task; VF = Verbal Fluency; SC = Symbol Coding. SCIL = Screener of Intelligence and Learning Disability.

⁎ Wald test *p* < .05.

⁎⁎ Wald test *p* < .01.

⁎⁎⁎ Wald test *p* < .001.

### 3.2 Executive functioning and symptomatology

Regression models of the PANSS-R, PHQ-9 and GAD-7 symptom measures showed that the TOL had an effect only on the GAD-7 (β = −0.13), while the BRIEF-A had an effect on all of them. We found no interaction effects between the TOL and BRIEF-A (range β = −0.06 to 0.08). Table 3 shows all results with the selected model. Sensitivity analyses showed that the results would not have been different if the TOL had been used dichotomously.

**Table 3:** Regression model of executive functioning on symptomatology.

	Model 1 (PANSS-R Total)		Model 2 (PHQ-9)		Model 3 (GAD-7)	
Parameter	β (SD)	Wald X^2^	β (SD)	Wald X^2^	β (SD)	Wald X^2^
Intercept	−0.04 (0.12)	0.12	0.14 (0.09)	2.40	0.14 (0.09)	2.11
Sex (male)	0.09 (0.14)	0.43	−0.16 (0.11)	1.96	**−0.24 (0.11)**	**4.45**⁎
Age (centered)	−0.01 (0.01)	1.63	**−0.01 (0.00)**	**4.38**⁎	**−0.02 (0.00)**	**18.98**⁎⁎
TOL	−0.02 (0.07)	0.06	0.01 (0.05)	0.08	**−0.13 (0.05)**	**6.77**⁎⁎
BRIEF-A	**0.32 (0.07)**	**20.34**⁎⁎⁎	**0.52 (0.06)**	**88.21**⁎⁎⁎	**0.55 (0.06)**	**95.46**⁎⁎⁎

TOL = Tower of London; BRIEF-A = Behavioral Rating Inventory of Executive Functioning – Adults; PANSS-R = Positive and Negative Symptom Severity – Remission; PHQ-9 = Patient Health Questionnaire – 9-item; GAD-7 = Generalized Anxiety Disorder – 7-item.

⁎ Wald test p < .05.

⁎⁎ Wald test *p* < .01.

⁎⁎⁎ Wald test p < .001.

### 3.3 Executive functioning and daily functioning

With the exception of the WHODAS mobility domain (β = −0.13), regression models showed no effect of the TOL on the WHODAS domains. There was an effect of the BRIEF-A on all the WHODAS domains. We found no interaction effects between the TOL and BRIEF-A (range β = 0.01 to 0.10). Results for all regression models with the selected model are shown in Table 4.

**Table 4:** Regression model of executive functioning on daily functioning domains.

	Model 1 (WHO-DAS - 1)		Model 2 (WHO-DAS - 2)		Model 3 (WHO-DAS - 3)	
Parameter	β (SD)	Wald X^2^	β (SD)	Wald X^2^	β (SD)	Wald X^2^
Intercept	0.05 (0.09)	0.28	**0.35 (0.10)**	**11.37**⁎⁎⁎	0.17 (0.10)	2.68
Sex (male)	−0.11 (0.11)	1.00	**−0.51 (0.13)**	**16.69**⁎⁎⁎	**−0.27 (0.13)**	**4.60**⁎
Age (centered)	0.00 (0.00)	0.02	**0.01 (0.01)**	**4.83**⁎	0.01 (0.01)	3.84⁎⁎
TOL	−0.07 (0.05)	2.02	**−0.13 (0.06)**	**5.43**⁎	−0.10 (0.06)	3.10
BRIEF-A	**0.54 (0.06)**	**94.18**⁎⁎⁎	**0.30 (0.06)**	**23.96**⁎⁎⁎	**0.22 (0.06)**	**12.06**⁎⁎⁎

TOL = Tower of London; BRIEF-A = Behavioral Rating Inventory of Executive Functioning – Adults; WHO-DAS = World Health Organization – Disability Assessment Schedule; WHODAS-1 = Domain Cognition; WHODAS-2 = Domain Mobility; WHODAS-3 = Domain Selfcare;

⁎ Wald test p < .05.

⁎⁎ Wald test *p* < .01.

⁎⁎⁎ Wald test *p* < .001.

## 4 Discussion

This study had two aims: to explore the differences between a performance-based measure and a self-report measure of executive functioning in a psychotic population, and to examine their associations with neurocognitive measures, symptom severity questionnaires and daily functioning items. We hypothesized that the performance-based measures would have an association with the neurocognitive measures, while the self-report measure would have an association with the symptomatology variables and the daily life outcomes.

We found that the performance-based measure had an association with the neurocognitive measures, and with the Screener for intelligence and learning disabilities. None of the performance-based measures were found to have an association with the symptomatologic variables, except with the anxiety screener. Lastly, while the performance-based measure had a small association with mobility, it had no association with the other daily-activity domains.

Except for a small association with motor function, the self-report measure had no associations with any of the neurocognitive measures or the screener for intelligence and learning disability. It was also associated with the different symptomatology variables and with all the different daily-activity domains. In all cases, the interaction between the performance-based measures and the self-report measure did not have any effect.

As expected, the performance-based executive-function measure and the self-report executive-function measure did not correlate with one another. One explanation for this lack of correlation may lie in the difference in psychometric qualities. While performance-based measures often have no association with daily-life activities and functional outcomes (Ardila, 2008; Wilson, 1993), they often reflect cognitive skills, because we often use them in a stimuli-poor environment such as a lab (Gohier et al., 2009), where they produce scores that are hard to generalize to the stimuli-rich environment that is daily life.

As self-report measures are influenced by emotional state, personality traits (Kajonius and Björkman, 2020; Buchanan, 2016), impairments in insight (Burton et al., 2016; Saperstein et al., 2012; Poletti et al., 2012) or metacognition (Butzbach et al., 2021; Ciurli et al., 2010) the scores they produce may also reflect either poor mood state or poor judgement of cognition, rather than just the cognitive performance in daily life. This is partly supported by the association that was found with the WHODAS. For the WHODAS, earlier research showed that the questionnaire might also reflect mood state rather than actual disability (Strassnig et al., 2018).Self-report measures might thus add ecological validity to performance-based measures (Gioia et al., 2010), while performance-based measures might add reliability to self-report measures (Isquith et al., 2014).

Another explanation for the lack of correlation between performance-based and self-report measures of executive functioning comes from research in various clinical groups, including people with ADHD. This suggests that performance-based tests measure processing efficiency and cognitive control, and thus reflect the technical quality of the executive functions. Our results support this idea, as they found an association between performance-based measures and neurocognitive measures. Because they are measured in a research-like setting, neurocognitive measures and learning abilities are both known to measure how well a person's cognitive system functions without the interference other people or distractions (Gohier et al., 2009; Patterson et al., 2001; Twamley et al., 2002). The reverse goes for the self-report measures, which provide insight into daily life (Gioia, 2008; Payne et al., 2011) and thus insight into the executive functions when there are distractions (Toplak et al., 2013; Nordvall et al., 2017). These distractions might lead to less efficient use of these functions, despite these functions being unimpaired in the lab, i.e. on performance-based measures. The less efficient use of the executive functions may explain why the self-report measure influences symptomatologic variables such as depression or anxiety, and also influences daily activities. If a person feels unable to successfully use the functions they need, it may make them anxious or depressed and vice versa. It may also lead them to believe that they have a poorer ability to perform daily activities. Most importantly, these results show that these two measures cannot and should not be used interchangeably to measure executive functioning. Instead, their use should be complementary.

### 4.1 Strengths and limitations

To the best of our knowledge, this is the first study to examine the difference between performance-based and self-report executive functioning in relation to different outcome variables in people with psychotic disorders. Although previous research found differences between the two forms of measurement (Toplak et al., 2013), also in people with a psychotic disorder (Bulzacka et al., 2013), their effect on the outcome variables that are important to recovery in psychosis has not been explored.

It is also a strength that this study was conducted in a large ongoing cohort. All participants were in mental healthcare, and had been diagnosed with a psychotic disorder. Demographic variables also showed that the cohort was representative of those currently in community mental healthcare in the Netherlands (Kortrijk et al., 2019). The study was set up with the help of a scientific board and a peer expert group. To attain the objectives stated in the study protocol (van Aken et al., 2021), both groups viewed, discussed and approved all the measures for use in this cohort.

Our study has three principal limitations. First, while we examined the influence of executive functioning on symptoms, the reverse may also have been of interest. The correlations between them are moderate, especially with regard to self-reported executive functioning, and anxiety and depressive measures. This was borne out in earlier research not concerned with psychosis, which suggested that anxiety and depression might influence the way participants score on self-report measures of executive functioning (Meltzer et al., 2017), rather than the other way around. In our case, this might mean anxiety and depressive symptoms may result in more difficulties using the executive functions successfully, as well as make a person feel that they cannot use the executive functions successfully.

Secondly, this study selected only a limited range of measures. Other measures of performance and self-report based executive functioning may have produced different results. For example, as a performance-based measure, the Wisconsin Card Sorting Task (WCST) is better equipped than the TOL to distinguish between clinical subgroups (Weyandt et al., 2014). Using the WCST thus might have resulted in a higher overlap with self-reported executive functioning. Similarly, it could be useful to further explore the influence of emotional state, insight and metacognition on self-report measures such as the BRIEF-A and the WHODAS, as the influence of either might explain the difference between the two measures. Rather than a difference in underlying construct, we might find a difference between cognitive performance and judgement of cognitive performance. Furthermore, it might also be we find the WHODAS to not be suited to measure disability properly. This needs further examination.

The third weakness concerns patients who were not willing to participate in this study, as there is still a group of people with a mental illness who are not in care. Although those with a high burden of disease – i.e., those with severe active symptoms – are of particular interest to this cohort, they are often unwilling or unable to participate. Even though our cohort study seems to be representative of the current Dutch outpatient population, nonrespondents would change its outcomes. Unfortunately, active psychosis often makes it difficult for this group to provide valid answers to a questionnaire such as the BRIEF-A. It is therefore possible that we will never be able to include clients who are in a severely psychotic episode.

In conclusion, our study confirms there is a difference between performance-based and self-reported executive functioning. Given their difference influences on neurocognitive measures, symptomatology and daily life activities, three hypotheses are possible: either psychometric qualities differ between the two measures, there is a difference between cognitive performance and judgement of cognitive performance, or the difference lies in the construct of executive functioning they measure. As we suggest, more research is needed to further test both ideas and to examine the causal relationships between executive functions and recovery measures. More research is also needed to examine the link over time between these two types of measure and outcome measures, and to explore their influence on recovery.

## CRediT authorship contribution statement

BvA has written this manuscript with the help of RR. This was under the direct guidance of AIW and CLM. All authors have commented on multiple versions of this manuscript. All authors have read and approved the final manuscript.

## Declaration of competing interest

The authors have no conflicts of interest to declare.
